# Observer variability in the assessment of CT coronary angiography and coronary artery calcium score: substudy of the Scottish COmputed Tomography of the HEART (SCOT-HEART) trial

**DOI:** 10.1136/openhrt-2014-000234

**Published:** 2015-05-19

**Authors:** Michelle C Williams, Saroj K Golay, Amanda Hunter, Jonathan R Weir-McCall, Lucja Mlynska, Marc R Dweck, Neal G Uren, John H Reid, Steff C Lewis, Colin Berry, Edwin J R van Beek, Giles Roditi, David E Newby, Saeed Mirsadraee

**Affiliations:** 1 University of Edinburgh/British Heart Foundation Centre for Cardiovascular Science, University of Edinburgh, Edinburgh, Lothian, UK; 2 Division of Cardiovascular and Diabetes Medicine, University of Dundee, Dundee, UK; 3 Edinburgh Heart Centre, Royal Infirmary of Edinburgh, Edinburgh, UK; 4 Borders General Hospital, Melrose, UK; 5 Centre for Population Health Sciences, University of Edinburgh, Edinburgh, UK; 6 British Heart Foundation Glasgow Cardiovascular Research Centre, University of Glasgow, Glasgow, UK; 7 Clinical Research Imaging Centre, University of Edinburgh, Edinburgh, UK; 8 Department of Radiology, Glasgow Royal Infirmary, Glasgow, UK

**Keywords:** CORONARY ARTERY DISEASE, CHEST PAIN CLINIC < CORONARY ARTERY DISEASE, IMAGING AND DIAGNOSTICS, CT SCANNING < IMAGING AND DIAGNOSTICS

## Abstract

**Introduction:**

Observer variability can influence the assessment of CT coronary angiography (CTCA) and the subsequent diagnosis of angina pectoris due to coronary heart disease.

**Methods:**

We assessed 210 CTCAs from the Scottish COmputed Tomography of the HEART (SCOT-HEART) trial for intraobserver and interobserver variability. Calcium score, coronary angiography and image quality were evaluated. Coronary artery disease was defined as none (<10%), mild (10–49%), moderate (50–70%) and severe (>70%) luminal stenosis and classified as no (<10%), non-obstructive (10–70%) or obstructive (>70%) coronary artery disease. Post-CTCA diagnosis of angina pectoris due to coronary heart disease was classified as yes, probable, unlikely or no.

**Results:**

Patients had a mean body mass index of 29 (28, 30) kg/m^2^, heart rate of 58 (57, 60)/min and 62% were men. Intraobserver and interobserver agreements for the presence or absence of coronary artery disease were excellent (95% agreement, κ 0.884 (0.817 to 0.951) and good (91%, 0.791 (0.703 to 0.879)). Intraobserver and interobserver agreement for the presence or absence of angina pectoris due to coronary heart disease were excellent (93%, 0.842 (0.918 to 0.755) and good (86%, 0.701 (0.799 to 0.603)), respectively. Observer variability of calcium score was excellent for calcium scores below 1000. More segments were categorised as uninterpretable with 64-multidetector compared to 320-multidetector CTCA (10.1% vs 2.6%, p<0.001) but there was no difference in observer variability.

**Conclusions:**

Multicentre multidetector CTCA has excellent agreement in patients under investigation for suspected angina due to coronary heart disease.

**Trial registration number:**

NCT01149590.

**KEY MESSAGES**

**What is already known about the subject?**

- CT coronary angiography can identify the presence of coronary artery disease with excellent diagnostic accuracy compared to invasive coronary angiography. Observer variability can influence diagnostic accuracy in cardiac CT.

**What does this study add?**

- In a large multicentre study, the observer agreement for the assessment of CT imaging was excellent. In addition, there was excellent agreement for the diagnosis of angina pectoris based on the combination of CT imaging and clinical assessment.

**How might this impact on clinical practice?**

- This study provides further support for the use of CT coronary angiography in the broad population of patients who present to the clinic for assessment of suspected angina due to coronary heart disease.

## Introduction

CT coronary angiography can identify the presence of coronary artery disease with excellent diagnostic accuracy as compared to invasive coronary angiography.^1^ The results of CT imaging are combined with clinical assessment in order to formulate an overall diagnosis on which to base management decisions. The impact of observer variability on the assessment of CT images and the subsequent diagnosis of angina pectoris due to coronary heart disease has potentially important implications for patient management.

The Scottish COmputed Tomography of the HEART (SCOT-HEART) trial is a randomised multicentre study that is assessing the role of CT coronary angiography in the assessment of patients with suspected coronary artery disease.^2^ Over 4000 participants assessed at the Rapid Access Chest Pain Clinic have been randomised to 64 or 320-multidetector CT coronary angiography plus standard care or standard care alone. This study will establish the additive diagnostic value of CT imaging in the management of these patients.

Good observer variability in the anatomical assessment of coronary artery stenosis by CT has previously been established.^3–6^ However, the most clinically relevant question relates to the observer variability in the final diagnosis of angina pectoris due to coronary heart disease. Moreover, differing scanner specifications and vendors, generalisability across multiple different sites, differing software applications and variations in clinic assessments are all potential sources of variability in the ultimate clinical assessment of such patients. Marked observer variability could lead to over or underdiagnosis of coronary artery disease, and lead to false reassurance or unnecessary further investigations or treatment.

The study aims were to assess observer variability in the assessment of stenosis severity and coronary artery calcification, to establish the observer variability of the diagnosis of angina pectoris due to coronary heart disease, and to compare observer variability between 64 and 320-multidetector CT scans in the SCOT-HEART trial.

## Methods

### Study design

The SCOT-HEART trial is a prospective multicentre randomised study of the role of CT coronary angiography in patients attending the rapid access chest pain clinic (NCT01149590,^2^). It has recruited 4146 patients from 12 sites, randomised 1:1 to CT coronary angiography plus standard care or standard care alone. The study was approved by the research ethics committee and all patients undertook written informed consent.

The images of 210 patients were assessed for this substudy. The first 50 scans at each site were coreported by the peripheral and central site to ensure a comparable approach and reporting quality. These scans were excluded from this substudy. In addition, patients with coronary artery bypass grafts or intracoronary stents were excluded from this substudy. Images were selected randomly and were selected to include a representative sample of study participants in terms of scanner type, presence of coronary artery disease on initial assessment and image quality. This meant that one-third of the 210 patients had no coronary artery disease, one-third had non-obstructive coronary artery disease and one-third had obstructive coronary artery disease based on the initial assessment of the CT imaging.

### CT imaging

CT imaging was performed using either 64 or 320-multidetector scanners (Aquilion ONE, Toshiba Medical Systems, Japan or Brilliance 64, Philips Medical Systems, the Netherlands) as described previously.^2^ Patients with heart rates >65 bpm received rate-limiting medication (intravenous metoprolol or oral ± intravenous metoprolol) and all patients received sublingual glyceryl trinitrate prior to imaging. Non-contrast imaging was performed to assess coronary artery calcification. Coronary angiography was obtained after the injection of iodinated contrast (Iomeron 400 (Bracco, UK) or Ultravist 370 (Bayer, USA) or Omnipaque 350 (GE Healthcare, USA). CT imaging using the 320-multidetector scanner was performed over a single heart-beat with wide volume imaging using an acquisition window of 70–80% or 30–80% depending on the heart rate. Imaging using the 64-multidetector scanner was performed using a ‘step and shoot’ technique with prospective gating or a helical technique with retrospective gating, depending on heart rate. Iterative reconstruction (Adaptive Iterative Dose Reduction or iDose4) was used to reconstruct images. Radiation dose was assessed using the dose length product, which was recorded from the scanner console after imaging.

### Image analysis

Images were assessed by observers blinded to the results of other assessments. Repeat assessments were performed at least 2 weeks apart in random order to prevent recall bias. Repeat assessment was performed by the same individuals as the initial assessment to assess intraobserver variability. A second set of observers assessed the images separately to assess interobserver variability.

Coronary artery calcium scoring was performed using dedicated software (VScore, Vital Images, Minnetonka, USA or scanner console software). Agatston score was calculated using a threshold of 130 HU (Hounsfield units) for each vessel and summed to give a total score.^7^

**Figure 1:**
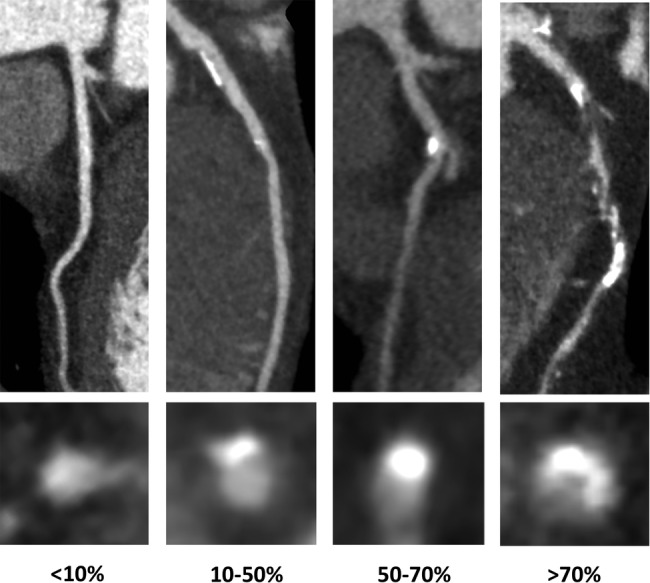
CT coronary angiography curved planar reformations and vessel cross sections showing lesions with different stenosis severity (none, <10%; mild, 10–49%; moderate, 50–70%; severe, >70%).

CT coronary angiogram images were assessed for stenosis severity on a segmental basis using a dedicated postprocessing workstation (Vitrea fX, Vital Images, Minnetonka, USA). A 15-segment model was used. Segments that were uninterpretable or absent were excluded. Luminal cross-sectional area was classified as normal (<10%) or having mild (10–49%), moderate (50–70%) or severe (≥70%) stenosis (figure 1).

On a per patient basis, the segmental CT coronary angiogram and coronary artery calcium score were used to decide the overall CT result. This assessment could be adjusted by the clinician based on any other relevant factors from the CT images (including image quality, stenosis length, plaque type, etc). Obstructive coronary artery disease was defined as a stenosis ≥70% in one or more major epicardial vessels or greater than 50% in the left main stem. Non-obstructive coronary artery disease was defined as coronary artery disease with stenosis >10% and not fulfilling the criteria for obstructive coronary artery disease.

The post-CT diagnosis of angina due to coronary heart disease was determined based on the CT result and the chest pain clinic assessment. Information available from the chest pain clinic included a history of coronary heart disease, exercise test result and the type of angina chest pain.^2^ ^8^ The post-CT diagnosis of angina pectoris due to coronary heart disease was classified as yes, probable, unlikely and no, and were dichotomised to assess the presence or absence of angina pectoris due to coronary heart disease.

### Image quality

Subjective image quality was assessed on a 4-point Likert scale (1 good scan quality, diagnostic, 2 moderate scan quality, diagnostic but suboptimal, 3 poor scan quality, limited diagnostic and 4 non-diagnostic scan quality). Vessels were also classified as interpretable or uninterpretable on a segmental basis.

Objective assessment of image quality was assessed by measuring CT attenuation and image noise, and calculating contrast-to-noise ratio. CT attenuation was measured in a region of interest in the ascending aorta at the level of the left main stem and liver of non-contrast scans and the ascending aorta at the level of the left main stem, liver and interventricular septum of contrast-enhanced scans. Image noise was determined as the SD of the HU in a region of interest drawn in the ascending aorta just above the level of the left main stem. Contrast-to-noise ratio was calculated as the attenuation in the aorta minus the attenuation in the liver and divided by the image noise in the aorta.

### Statistical analysis

Statistical analysis was performed using PASWStatistics (V.18 for Mac, IBM) and Graphpad Prism (V.6 for Mac). Interobserver and intraobserver variabilities were assessed using κ statistics and Bland-Altman plots. A Cohen's κ statistic of less than 0.2 indicated poor agreement, 0.21–0.4 fair agreement, 0.41–0.6 moderate agreement, 0.61–0.8 good agreement and 0.81–1 excellent agreement. Normally distributed quantitative variables are presented with mean and 95% CI. Non-normally distributed data are presented as median and IQR. Statistical significance was assessed using analysis of variance, student's t test or Pearson's χ^2^ test as appropriate. A statistically significant difference was defined as a two-sided p value <0.05.

## Results

We assessed 210 patients (62% male) who had a mean body mass index (BMI) of 29 (95% CI 28 to 30) kg/m^2^ and mean heart rate of 58 (95% CI 57 to 60)/min (table 1). Arrhythmia was uncommon, with 3.3% of the patients having ectopic beats and 1% atrial fibrillation.

**Table 1:** Demographic details

Parameter	
N	210
Age (years)	58 (57, 60)
Male	130 (62%)
Body mass index (kg/m^2^)	29 (28, 30)
64/320 multidetector scanner	72 (34%)/138 (66%)
Previous history of coronary artery disease	18 (9%)
Coronary artery calcium score (Agatston units)	373 (242, 505)
Zero coronary artery calcium score	142 (68%)
CT overall assessment	
No coronary artery disease	70 (33%)
Non obstructive coronary artery disease	70 (33%)
Obstructive coronary artery disease	70 (33%)
CT vessels with significant coronary artery disease	
One vessel disease	39 (19%)
Two vessel disease	21 (10%)
Three vessel disease	10 (5%)

(Mean and (95% CI) or median (IQR) or number (percentage).).

### Coronary artery disease and angina

There was excellent or good intraobserver and interobserver agreement in the assessment of CT imaging for the presence or absence of coronary artery disease: 95% agreement, κ 0.884 (95% CI 0.817 to 0.951) and 91% agreement, κ 0.791 (95% CI 0.703 to 0.879), respectively (table 2). Similar agreement was also seen for the presence or absence of angina pectoris due to coronary heart disease: 93% agreement, κ 0.842 (95% CI 0.918 to 0.755) and 86% agreement, κ 0.701 (95% CI 0.799 to 0.603), respectively (table 2).

**Table 2:** Intra and inter observer variability for (A) the presence of coronary artery disease on CT imaging and (B) the diagnosis angina pectoris due to coronary heart disease after CT imaging

Intra-observer			Intra-observer		
	Absent	Present		Absent	Present
(A) Coronary artery disease on CT					
Absent	67	3	Absent	57	13
Present	8	132	Present	6	134
(B) Angina pectoris due to coronary heart disease					
Absent	130	3	Absent	113	10
Present	12	65	Present	20	67

When the overall CT result was classified as obstructive, non-obstructive or no coronary artery disease on a per patient basis (table 3), there was excellent intraobserver agreement (87% agreement, κ 0.807 (95% CI 0.876 to 0.738)) and good interobserver agreement (81% agreement, κ 0.721 (95% CI 0.799 to 0.643)). Agreement was highest for scans with no coronary artery disease as compared to non-obstructive or obstructive coronary artery disease for both intraobserver variability (96% vs 96% and 83%, respectively) and interobserver variability (81% vs 86% and 77%, respectively).

**Table 3:** Intraobserver (A) and interobserver (B) variability in per patient CT assessment

	None	Non-obstructive	Obstructive
Panel (A)			
None	67	2	1
Non-obstructive	7	58	5
Obstructive	1	11	58
Panel (B)			
None	57	12	1
Non-obstructive	4	60	6
Obstructive	2	14	54

Using a 4-point scale to assess the presence of angina pectoris secondary to coronary heart disease (yes, probable, unlikely and no; table 4), there was good. intraobserver variability (κ 0625 (95% CI 0.709 to 0.541)) and moderate interobserver variability (κ 0.497 (95% CI 0.581 to 0.413)). Similarly, based on a 4-point scale (none, mild, moderate, severe), there was moderate intraobserver and interobserver variability in the per segment assessment of stenosis severity: κ 0.521 (95% CI 0.490 to 0.552) and κ 0.459 (95% CI 0.428 to 0.490), respectively (table 5).

**Table 4:** Intraobserver (A) and interobserver (B) observer variability in per segment CT assessment of stenosis severity

	Yes	Probable	Unlikely	No
Panel (A)				
Yes	42	5	2	5
Probable	10	8	1	4
Unlikely	0	0	15	17
No	1	2	3	95
Panel (B)				
Yes	34	16	3	1
Probable	9	8	5	1
Unlikely	1	9	17	5
No	4	6	14	77

**Table 5:** Intraobserver (A) and interobserver (B) variability in per segment CT assessment of stenosis severity

	<10%	10–49%	50–70%	>70%
Panel (A)				
<10%	2054	142	15	13
10–49%	112	171	35	13
50–70%	43	55	43	16
>70%	24	35	26	89
Panel (B)				
<10%	1778	234	19	23
10–49%	91	182	33	17
50–70%	24	70	32	22
>70%	19	44	30	75

### Coronary artery calcium score

**Figure 2:**
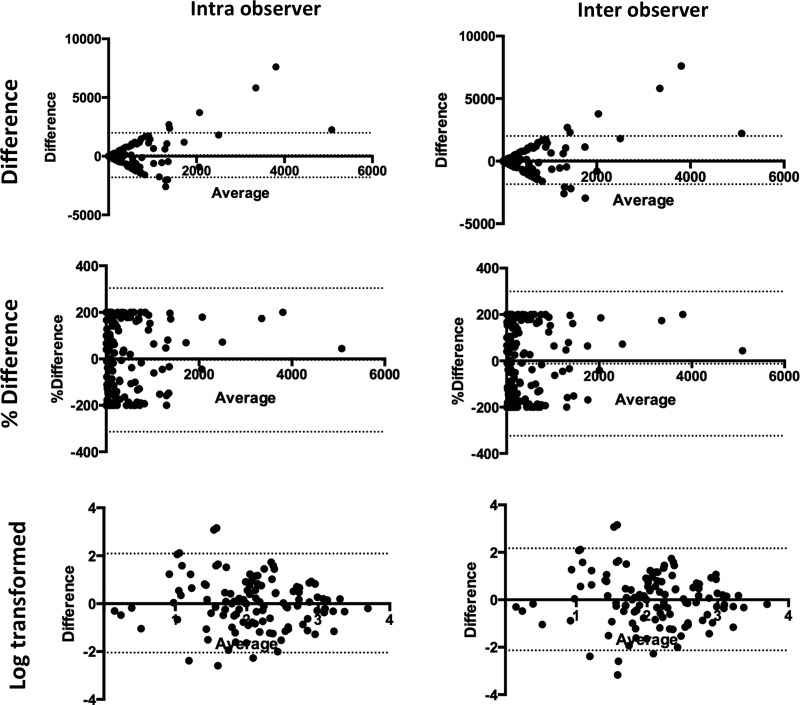
Bland-Altman plots for intra and inter observer variability for the assessment of total Agatston score (dotted lines represent the limits of agreement).

**Figure 3:**
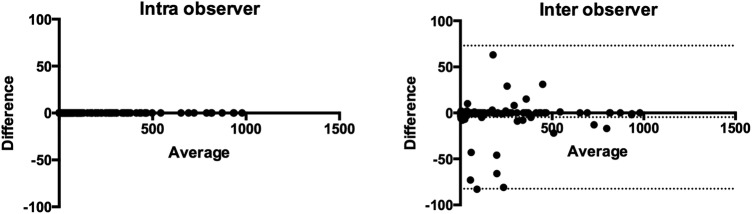
Bland-Altman plots for intra and inter observer variability for the assessment of total Agatston score for patients with a calcium score less than 1000 (one outlier was excluded from the inter observer variability assessment, dotted lines represent the limits of agreement).

There were no differences in Agatston calcium score on intraobserver or interobserver assessment (373 (95% CI 224 to 505) Agatston units versus 278 (95% CI 202 to 354) Agatston units, p=0.138 and 290 (95% CI 210 to 370) Agatston units, p=0.191). Bland-Altman plots showed that the level of calcification systematically affected intraobserver and interobserver variability of the Agatston score (figure 2) with both intraobserver and interobserver variability increasing as the calcium score increased. However, for patients with calcium score of less than 1000, the intraobserver and interobserver was excellent (figure 3).

### Image quality

The number of segments that were categorised as uninterpretable was fivefold higher using 64 as compared to 320-multidetector scanners (10.1% vs 2.6%, p<0.001). There was no difference in mean aorta contrast attenuation between 64 and 320-multidetector scanners, but there was a small but significant reduction in image noise in contrast-enhanced 320-multidetector CT (table 6). Radiation dose was higher for 64 as compared to 320-multidetector CT (411.46 (95% CI 344.91 to 478.0) vs 314.62 (95% CI 228.10 to 347.14), p 0.01) Interobserver variability for 64 and 320-multidetector CT were similar, with a large overlap of CIs, for the assessment of coronary artery disease (κ 0.729 (95% CI 0.596 to 0.862) vs 0.717 (95% CI 0.619 to 0.815) respectively) and the post-CT diagnosis of angina due to coronary heart disease (κ 0. 0.518 (95% CI 0.416 to 0.620) and 459 (95% CI 0.314 to 0.604), respectively).

**Table 6:** Quantitative assessment of image quality with 64 and 320 multidetector CT

	320 MDCT	64 MDCT	p Value
*Attenuation*			
Non contrast			
Aorta	60	38	**<0**.**001**
Liver	52	49	0.085
CTCA			
Aorta	500	495	0.801
Septum	85	138	**<0**.**001**
Liver	62	66	0.466
*Noise*			
Non contrast			
Aorta	19	20	0.564
Liver	34	31	**0**.**013**
CTCA			
Aorta	37	40	**0**.**014**
Septum	34	41	**<0**.**001**
Liver	40	41	0.558
*CNR*			
Non contrast			
Aorta	19	20	0.564
Liver	34	31	**0**.**013**
CTCA			
Aorta	37	40	**0**.**014**
Septum	34	41	**<0**.**001**
Liver	40	41	0.558

Bold represents statistically significant values.

CNR, contrast to noise ratio; CTCA, CT coronary angiography; MDCT, multidetector CT.

## Discussion

This study confirms the excellent intraobserver and interobserver agreement for the assessment of the presence or absence of coronary artery disease by CT coronary angiography. In addition, for the first time, we establish the excellent interobserver and intraobserver agreement for the post-CT diagnosis of the presence or absence of angina pectoris due to coronary heart disease. This study provides a basis for the clinical use of CT coronary angiography to guide the diagnosis, management and treatment of patients being assessed for suspected angina due to coronary heart disease.

In keeping with previous studies, we have shown the excellent agreement for the per patient assessment of CT coronary angiography.^3–6^ Observer variability and diagnostic accuracy in CT coronary angiography may be influenced by patient factors such as the presence of heavily calcified coronary artery disease or a rapid heart rate. Calcified atherosclerotic plaque may be overestimated on CT imaging due to a combination of ‘beam hardening’ and ‘blooming’ artifacts. The assessment of calcified plaque is therefor associated with higher observer variability than non-calcified plaque.^9^ ^10^ In particular eccentric calcification is associated with the highest observer variability in the assessment of stenosis severity.^3^ However, Ovrehus *et al*^11^ found that for patients with an Agatston score below 400 Agatston units, the calcium score was not a predictor of observer variability in the assessment of stenosis severity.

In keeping with previous studies,^12^ we showed that observer variability in calcium scoring increases as the Agatston score increases. However, for patients with a calcium score below 1000 Agatston units, the observer agreement was excellent. In addition to good intraobserver and interobserver variability in calcium scoring, good interscan variability has also been established.^13^ However, differences in calcium scores have been established in phantom studies between different scanners.^14^ Therefore, it is important that studies assessing the progression of calcium scoring take scanner variations into account. The excellent observer agreement identified in this study supports its application in the assessment of cardiovascular risk.

The prevalence of coronary artery disease in a population can influence the results of studies of non-invasive imaging techniques. Nicol *et al* showed that there was the best observer agreement for patients with a low to intermediate pretest probability of coronary artery disease as compared to patients with a higher pretest probability. Previous studies of observer variability in populations with a high prevalence of coronary artery disease showed slightly poorer observer agreement as compared to studies with a lower prevalence of coronary artery disease^3^ ^5^ ^6^ ^11^ ^15^ (see online supplementary table S1). This is likely to be due to the higher prevalence of heavily calcified vessels in patient populations with a higher risk of cardiovascular disease. In our study, we identified slightly higher observer agreement for patients with no coronary artery disease as compared to non-obstructive or obstructive coronary artery disease. Unlike other studies we did not exclude patients based on heart rate, the presence of arrhythmias, body mass index or weight. Our study assessed patients with a representative prevalence of coronary artery disease in those presenting to the clinic for assessment of suspected angina due to coronary heart disease. Therefore our results regarding observer variability can be directly transferred to the assessment of such patient populations.

**Figure 4:**
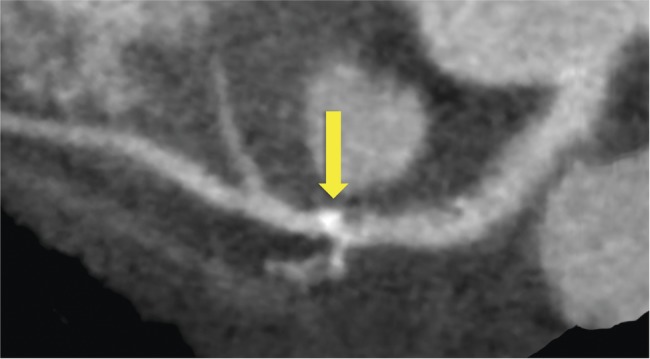
CT coronary angiography curved planar reconstruction of the left anterior descending coronary artery showing an atherosclerotic plaque with calcified and non-calcified components. The location of this plaque, which spans the origin of the first diagonal vessel, can cause differences in segmental classification between observers as it could be classified as proximal left anterior descending artery, mid left anterior descending artery, or both.

**Figure 5:**
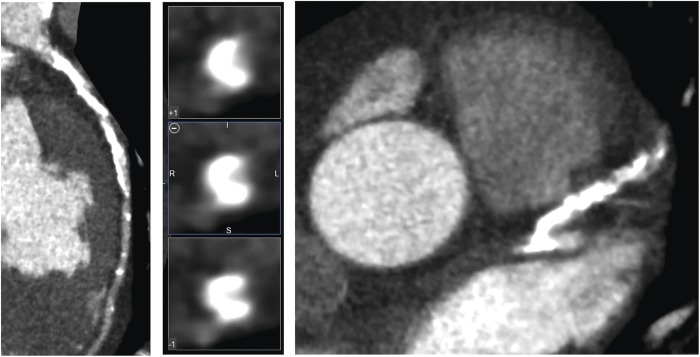
CT coronary angiography images of a heavily calcified left anterior descending artery. The blooming artifact from such heavily calcified plaque can lead to differences in observer classification of stenosis severity.

There was excellent agreement for the overall diagnosis of the presence or absence of angina pectoris due to coronary heart disease in this study. However, when more categories are included in an assessment, the level of agreement understandably decreases. On a per segment basis, differences in anatomical classification are an important source of observer variability. For example, figure 4 shows an atherosclerotic plaque in the left anterior descending artery at the branch of the first diagonal. This could be classified as either in the proximal or mid left anterior descending artery segments. In addition, lesions of borderline significance may be up or down graded depending on the observer as shown in figure 5. The choice of cut-off value for the definition of stenosis severity may also affect observer variability. Most previous studies have assessed observer variability for the identification of any atherosclerotic plaque or the assessment of stenoses greater than 50%. Previous studies identified inter-observer agreement for the presence of stenosis greater than 50% of between 81% and 96%, with κ scores between 0.66 and 0.91 (see online supplementary table S1).^3^ ^5^ ^6^ ^11^ ^15^ Similar to our study, when using a cut-off of greater than 70%, Nicol *et al*^3^ identified interobserver agreement of 87% on a per patient basis, 97% on a per vessel basis and 99% on a per segment basis. The use of the 70% cut-off for the diagnosis of obstructive coronary artery disease in our study was felt to be the most clinically relevant parameter for the assessment of CT imaging.

Different scanner types and CT coronary angiography protocols may affect observer variability. In a study of patients undergoing 64-multidetector CT coronary angiography, there was good interobserver agreement for prospectively gated scans and excellent interobserver agreement for retrospectively gated scans (κ 0.724 and 0.823, respectively).^16^ In a study of 320-multidetector CT, the per patient interobserver variation for the detection of significant coronary artery disease (≥50% stenosis) was good (κ 0.72 (95% CI 0.62 to 0.81). A study of the diagnostic accuracy of dual source CT identified good interobserver variability with a κ score of 0.79.^17^ In our study, there were more non-diagnostic segments with 64 as compared to 320-multidetector CT. Khan *et al*^18^ showed that there were 0.1% non-diagnostic segments with 320-multidetector as compared to 3.3% with 64-multidetector CT coronary angiography. However, we have shown that despite this difference, intraobserver and interobserver variability was similar between the two scanner types. The excellent observer variability identified in this study is therefore important for the large-scale adoption of this technique for patients with suspected coronary artery disease.

This study includes a representative sample of image quality and severity of coronary artery disease selected randomly from a large multicentre study. However, this meant that the effect of image quality on observer variability could not be assessed due to low numbers in the poor image quality group. In addition, we included a representative proportion of scans from each scanner, so there are not equal numbers in both groups. This study could not assess diagnostic accuracy, as not all patients went on to have invasive coronary angiography. The post CT diagnosis of angina due to coronary heart disease was based only on CT coronary angiography and the rapid access chest pain clinic assessment. The assessment of myocardial perfusion was not performed in this study. However, we did have broad inclusion criteria and included a representative population of patients attending the clinic that included patients with arrhythmia and nearly a half of patients had a body mass index >30 kg/m^2^. In our study the intraobserver agreement for the diagnosis of coronary artery disease was 95% and the interobserver agreement was 91%, similar to previous studies (see online supplementary table S1). However, this means that there were disagreements in 5% for intraobserver assessment and 9% for interobserver agreement for the diagnosis of coronary artery disease. Many of these cases were in patients with reduced image quality. Nevertheless, the potential for disagreement in the assessment of CT coronary angiography must be remembered when this technique is applied in a clinical setting.

In conclusion, we have established the excellent observer agreement for the assessment of coronary artery calcium score, CT coronary angiography assessment of stenosis severity and the post CT diagnosis of angina pectoris due to coronary heart disease. This supports the use of CT coronary angiography in the assessment of patients with suspected angina due to coronary heart disease.
